# Parallel Multi-Gene Panel Testing for Diagnosis of Idiopathic Hypogonadotropic Hypogonadism/Kallmann Syndrome

**DOI:** 10.1155/2019/4218514

**Published:** 2019-10-27

**Authors:** Manickavasagam Senthilraja, Aaron Chapla, Felix K. Jebasingh, Dukhabhandhu Naik, Thomas V. Paul, Nihal Thomas

**Affiliations:** Department of Endocrinology, Diabetes & Metabolism, Christian Medical College, 632 004 Vellore, India

## Abstract

Kallmann syndrome (KS)/Idiopathic hypogonadotropic hypogonadism (IHH) is characterized by hypogonadotropic hypogonadism and anosmia or hyposmia due to the abnormal migration of olfactory and gonadotropin releasing hormone producing neurons. Multiple genes have been implicated in KS/IHH. Sequential testing of these genes utilising Sanger sequencing is time consuming and not cost effective. The introduction of parallel multigene panel sequencing of small gene panels for the identification of causative gene variants has been shown to be a robust tool in the clinical setting. Utilizing multiplex PCR for the four gene KS/IHH panel followed by NGS, we describe herewith two cases of hypogonadotropic hypogonadism with a Prokineticin receptor 2 (PROKR2) gene and KAL1 gene mutation. The subject with a PROKR2 mutation had a normal perception of smell and normal olfactory bulbs on imaging. The subject with a KAL1 gene mutation had anosmia and a hypoplastic olfactory bulb.

## 1. Introduction

Idiopathic hypogonadotropic hypogonadism is associated with normosmia. Kallmann syndrome (KS) is a unique disease phenotype of idiopathic hypogonadotropic hypogonadism (IHH) associated with anosmia [1]. More than 15 genes have been implicated in the pathogenesis of KS/IHH [2].

Molecular genetic testing of the known genes would not only provide a confirmed diagnosis of KS/IHH but also help in understanding the underlying molecular mechanisms. Sequential screening of the KS/IHH genes using Sanger sequencing has been the gold standard for genetic diagnosis [3]. However, these methods are difficult to utilize, particularly in relation to diseases like KS/IHH with similar clinical features owing to the large number of genes, high cost, as well as prolonged cycles of sequential testing. Parallel multi-gene testing (next generation sequencing-NGS) with its high-throughput sequencing capability can handle multiple genes related to genetic disorders simultaneously [4]. This may aid in the differential diagnosis for diseases associated with known mutation sites and may give a clue to novel pathogenic mechanisms.

### 1.1. Case-A1

A 22 year old gentleman presented with poorly developed secondary sexual characters, born at term to non-consanguineous parents, with normal developmental milestones and intelligence on par with age. There was no history of seizures, blurring of vision, colour blindness, hearing loss, or movement disorder. He had received intramuscular testosterone injections for 4 years prior to presentation. He was the eldest of 8 siblings (two brothers and six sisters), with an unremarkable family history.

His weight was 63 kg, height was 185 cm, upper and lower segment ratio of 0.7(87 cm/98 cm). He had a high pitched voice, intact olfactory perception, absent facial and axillary hair, pubic hair (Tanner's stage 3), with bilateral descended prepubertal testes (2 ml in volume), stretched penile length of 7 cm and neurologic examination was essentially normal.

On biochemical investigation, he was found to have an LH-0.69 mIU/ml (N-0.8–7.6 mIU/ml), FSH-0.77 mIU/ml (N-0.7–11.1 mIU/ml), Testosterone-103 ng/dl (N-270–1030 ng/dl), suggestive of hypogonadotropic hypogonadism. Other hormonal axes were normal and magnetic resonance imaging of the brain including the olfactory bulb was normal.

### 1.2. Case-A2

**Figure 1:**
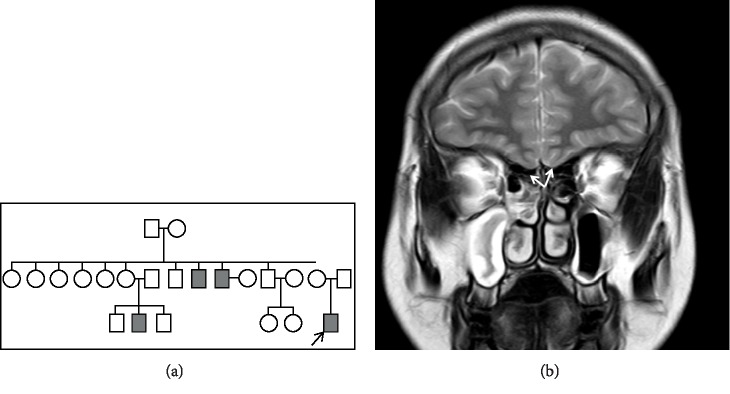
(a) Pedigree chart of Case-A2. (b) Coronal T2 weighted image showing bilateral hypoplastic olfactory bulbs.

An 18 year old boy presented with absent pubertal development. There was no perception of smell; there was no hearing loss or involuntary movements. There was a history of probable hypogonadism in the family with two paternal uncles and one cousin (Figure 1(a)). The height was 163 cm, arm span was 172 cm, upper and lower segment ratio of 0.75, consistent with eunachoid body proportions.There was no facial and body hair, the stretched penile length was 8 cm, the testes were infantile (1 ml) and neurological examination was normal. Laboratory tests confirmed hypogonadotropic hypogonadism with low testosterone-115 ng/dl (N-270–1030 ng/dl), low FSH-0.85 mIU/ml (N-0.7–11.1 mIU/ml) and low LH-0.74 mIU/ml (N-0.8–7.6 mIU/ml) levels. MRI of the brain displayed a hypoplastic olfactory bulb consistent with Kallmann syndrome (Figure 1(b)).

## 2. Mutational Analysis

Genetic testing was carried out for Kallmann syndrome using parallel multigene panel testing targeting four genes KAL1, FGFR1, PROKR2, and PROK2. As a pilot study we had designed this restricted gene panel to include genes which are more frequently implicated in majority of the cases of IHH/KS.

Genomic DNA was extracted using the Qiagen gentra kit method, followed by multiplex PCR based target enrichment for the four genes. A well established and cost-effective multiplex PCR coupled parallel multigene panel testing in the clinical setting has been followed [4]. In short the multiplex PCR consisted of 33 amplicons covering 23,435 bp which were pooled and processed for shearing, barcoded adaptor ligation and size selection to yield a library with a 200 bp insert. The library was amplified using the Ion one touch emulsion PCR followed by the Ion one touch enrichment to remove the excess of ion beads. The template was loaded on the 314 or 316 ion chips for sequencing on the Ion torrent Personal Genome Machine (PGM) sequencer. With this protocol we were able to sequence the samples with >1000× mean coverage and achieve 100% of the target with a minimum coverage of 20× as shown in Table 1. Therefore, the samples were sequenced without any gaps in the coding or the splice site regions.

## 3. Results

**Figure 2:**
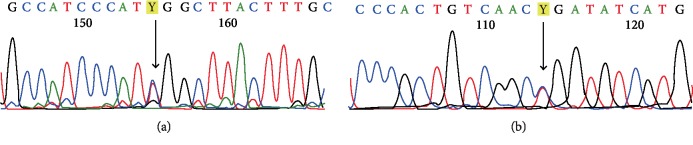
(a) PROKR2 c.563>T (p.Ser188Lys). (b) KAL1 c.1369C>T (p.Arg457Ter).

Subject A1 was found to have a heterozygous reported mutation c.563C>T, p.Ser188Lys in the Prokineticin receptor 2 (PROKR2) gene (Figure 2(a)). Cole et al., has reported this mutation in a male patient with normosmic hypogonadotropic hypogonadism [5]. This mutation has been further confirmed by Sanger sequencing.

Subject A2 was found to be positive for a reported homozygous KAL1 gene mutation c.1369C>T resulting in a change in amino acid arginine at codon 457 to a termination codon (p.Arg457Ter) (Figure 2(b)). Oliveira et al., has reported the same mutation of KAL1 gene in a male patient with cryptorchidism with hypogonadism [11].

Sanger confirmation data for the variants identified by NGS is shown in Figures 2(a) and 2(b).

## 4. Discussion

In our study, we identified the mutation of PROKR2 in subject A1, who had normosmic hypogonadotropic hypogonadism. PROK2, a peptide precursor and its receptor PROKR2, was considered to be a promising candidate gene for KS/IHH. Even though PROK2/PROKR2 is inherited in an autosomal recessive pattern, the PROK2/PROKR2 mutation was almost always found in the heterozygous state. Recent evidence suggests that an oligogenic inheritance may in part account for this observation. Our patient (subject A1) also found to have a heterozygous mutation in PROKR2[c.563C>T, p.Ser188Lys]. Consistent with our findings, Cole et al., has reported the same heterozygous PROKR2 mutation of Ser188Lys in the transmembrane domain in a male patient with IHH [5].

We identified a homozygous nonsense mutation (c.1369C>T; p.Arg457Ter) of KAL1 in subject A2 who presented with anosmic hypogonadotropic hypogonadism. The Kallmann syndrome 1 (KAL-1), a candidate gene for X-linked KS encodes a protein (anosmin) which is involved in neuronal migration and axonal pathfinding of GnRH neurons. Mutations in KAL1 are mainly nonsense mutations, frame shift mutations or large gene deletions [6]. Our subject had a non-sense mutation leading to the formation of a terminator codon.

Kallmann syndrome (KS) is a genetically heterogeneous condition defined by hypogonadotropic hypogonadism (HH) and anosmia where as Idiopathic hypogonadotropic hypogonadism (IHH) is not associated with anosmia. It can be associated with other developmental anomalies such as cleft lip or palate, dental agenesis, ear anomalies, congenital hearing impairment, renal agenesis, bimanual synkinesis or skeletal anomalies [7]. Causative genes for Idiopathic hypogonadotropic hypogonadism (IHH) with anosmia includes: KAL1, FGFR1, FGF8, PROKR2 PROK2, CHD7, HS6ST1, SOX10, SEMA3A, WDR11, IL17RD, and FEZF139 [2]. Genes involved in IHH that are associated with a normal sense of smell and include GNRHR, GNRH1, KISS1R, KISS1, TACR3, and TAC3 [8]. Incomplete penetrance and variable expressivity of the disease among patients with identical mutations can be observed for most KS/IHH genes [9].

The challenge for the genetic diagnosis of KS/IHH using sanger sequencing is genetic heterogeneity and oligogenic inheritance. In such cases, the targeted NGS can support the genetic diagnosis of hypogonadotropic hypogonadism with genetic heterogeneity and oligogenic inheritance [3].

Therefore, this approach may explain why KS/IHH subjects sharing an identical mutation exhibit different phenotypes [10]. With the multiplexing option, NGS has shown to be an alternative cost effective tool for screening single genes and small gene panels in a clinical setting [12]. Further, the identified mutation can be screened in the family members and also provide genetic counseling.

In summary, multiplex PCR coupled with NGS is a rapid and cost effective tool for identifying mutations of hypogonadotropic hypogonadism. These small gene panels are flexible and robust tools in screening heterogenous disorders like hypogonadotropic hypogonadism.

## Figures and Tables

**Table 1:** Ion torrent coverage analysis report.

Target base coverage	
Bases in target regions	23,435
Average base coverage depth	1,145
Target base coverage at 20×	100.00%
Target base coverage at 100×	96.60%
